# Predictors and Outcomes of Falls in Older Adults Presenting to the Emergency Room in Saudi Arabia: A Cross-Sectional Analysis

**DOI:** 10.7759/cureus.47122

**Published:** 2023-10-16

**Authors:** Abdulsalam Aleid, Hadeel K Bin Shuiel, Nouf A Alyabis, Anwar H Alfaraj, Dana J Dahlan, Fawaz M Alkhatib, Muteb N Alotaibi, Khalid N Almulhim, Abbas Al Mutair

**Affiliations:** 1 Neurosurgery, King Faisal University, Al Ahsa, SAU; 2 Emergency Medicine, College of Medicine, Alfaisal University, Riyadh, SAU; 3 Emergency Medicine, College of Medicine, Dar Al-Uloom University, Riyadh, SAU; 4 Emergency Medicine, Batterjee Medical College, Jeddah, SAU; 5 Emergency Medicine, College of Medicine, Umm Al-Qura University, Makkah, SAU; 6 Surgery, King Faisal University, Hofuf, SAU; 7 Research Center, Almoosa Specialist Hospital, Al Ahsa, SAU

**Keywords:** risk factors for falls, quality of emergency care, chronic disease and falls, environmental safety fall-related fractures, emergency room visits, fall incidents in saudi arabia

## Abstract

Introduction: Saudi Arabia is witnessing a demographic shift characterized by a rising elderly population. Cases of fall in this demographic have emerged as a significant health concern, especially in emergency room (ER) settings. Despite this, there is limited research on the causes and outcomes of such incidents. This study intends to bridge the gap in understanding the factors leading to falls in elderly patients presenting to ERs and the subsequent outcomes. Such understanding is pivotal for the formulation of effective prevention strategies and enhanced healthcare services for the elderly.

Methods: To achieve the study's objectives, we employed SPSS software for Windows, version 28.0 (IBM Corp., Armonk, NY) for data analysis. We collected demographic information, including age, gender, education, employment status, and location, to measure patient satisfaction with the quality and responsiveness of emergency care, using Likert scale responses via electronic survey conducted as a cross-sectional study from January 2023 to August 2023, summarizing it using descriptive statistics. We analyzed categorical variables by frequencies and percentages. Chi-square tests were utilized to examine differences in distribution across categories for fall factors, and a p-value below 0.05 was deemed significant. Through logistic regression, we pinpointed the predictors of falls among older adults, showcasing the strength and direction of these relationships. Adjusted odds ratios with 95% confidence intervals were documented. A perception survey was also conducted to evaluate ER patient satisfaction.

Results: Our results shed light on various aspects of fall prevention and emergency care. There was a pronounced representation in age groups of 18-24 and 25-34 years, indicating the need for interventions tailored to different age groups. Patterns were identified where subjects engaged in limited physical activity and consumed alcohol infrequently. Mobility and balance problems were commonly found, stressing the need to address these issues. Chronic conditions such as hypertension and diabetes correlated with fall incidents. Additionally, sociodemographic factors like gender, education, and employment status played a role in influencing the risk of falls. Although age and location seemed to have a less pronounced effect, there exists an opportunity to enhance communication and patient participation in emergency care for improved experiences.

Conclusion: The findings from our study provide crucial insights into the prevention of falls and enhancement of emergency care for Saudi Arabia's elderly population. By revealing the intricate relationships between sociodemographic attributes, health indicators, chronic ailments, and incidents of falls, we emphasize the need for well-rounded interventions. There is a pressing requirement for comprehensive fall prevention initiatives tailored to specific risk groups. Additionally, improving ER services is integral to ensuring the safety and well-being of older adults. This research can serve as a foundational resource for healthcare professionals and policymakers to devise robust strategies to reduce fall-related injuries and elevate the quality of emergency care outcomes.

## Introduction

Falls are a significant concern for the elderly worldwide and a common presentation in emergency departments. Defined by the World Health Organization (WHO) as an event leading an individual to rest unintentionally on a lower level, falls have become the second leading cause of death globally [1]. Notably, 684,000 individuals succumb to fall-related injuries every year, with over 80% of these incidents occurring in low- and middle-income countries [1]. In the context of the elderly, defined by the United Nations as those aged 60 years and older [2], various risk factors contribute to falls. These range from age-related changes in the nervous system affecting balance and gait coordination to medications such as antihypertensives, sedatives, and more [3-6]. Socioeconomic status, environmental factors, comorbidities, and even gender also play a role, with studies suggesting a higher propensity for falls among females [7]. The consequences of these falls are multifaceted, from physical injuries like hip fractures [4] to mental and socioeconomic implications like depressive symptoms and financial losses [5,8,9]. Falls are expansive, and prevention is pivotal; increased physical activity, adequate sleep, and cautious medication management are usually emphasized [10]. In the context of Saudi Arabia, this concern is magnified as the nation witnesses a demographic shift towards an aging population [5,8,9]. Still, localized research, especially pertaining to emergency room (ER) presentations, remains limited. Thus, this study endeavors to explore predictors of falls among Saudi's elderly, the outcomes of these incidents, and their broader implications, all while testing specific hypotheses concerning chronic health conditions, medication use, and demographic associations with fall outcomes.

## Materials and methods

This study assessed older adults presenting to emergency rooms in various provinces of the Saudi Arabia, encompassing Middle Eastern, Northern, South, and Western provinces. Conducted as a cross-sectional investigation from January to August 2023, the focus was primarily on individuals aged 60 years or older who were residents of Saudi Arabia. From the targeted segment, 1,763 participants completed our structured questionnaire, which was the primary data collection tool. This questionnaire shed light on demographic details, chronic health conditions, medication consumption, and fall history. With the intention of maintaining rigor, non-residents, those who declined participation, individuals with cognitive impairments, or previous participants were excluded.

Our study variables encapsulated independent factors, including demographics and health conditions, and dependent factors like chronic condition prevalence and medication-related issues within the participants. For precision in our approach, a pilot study was executed ahead of the main data collection, ensuring the reliability and validity of our instruments. Study approval was obtained from the Research Ethics Committee, King Faisal University, Ahsa, Saudi Arabia (approval no. KFU-REC-2023-SEP-ETHICS1,147), ensuring adherence to ethical guidelines, including respect for participants' rights, confidentiality, and voluntary participation.

IBM SPSS software for Windows, version 28.0 (IBM Corp., Armonk, NY) was used for data analysis. Our statistical analyses employed both descriptive and inferential methods. We used descriptive statistics such as means, medians, and percentages to shed light on demographics and primary variables. In contrast, inferential tests like chi-square tests and logistic regression were utilized to assess variable associations and pinpoint significant differences. We established our benchmark for statistical significance at p < 0.05 and determined association strengths using calculated confidence intervals.

## Results

Sociodemographic characteristics

A total of 1,763 participants completed the survey; their demographics are shown in Table 1. It presents data on age groups, with the majority falling within the 18-24 (33.0%) and 25-34 (19.3%) age ranges. Gender distribution was almost evenly split, with 59.7% females and 40.3% males. In terms of education level, 69.0% had a bachelor’s degree, while 18.0% had high school education or less. Employment status revealed that 37.2% were employed full-time, and 30.9% were students. Geographically, 91.5% resided in urban areas. This comprehensive overview of the demographic variables provides valuable insights into the characteristics of the study population.

**Table 1:** Sociodemographic characteristics of study participants The data presented in the table is based on a sample of adult participants and may not represent the entire population. Percentages may not add up to exactly 100% due to rounding. Age categories "Under 18" and "Above 65" have relatively smaller counts and should be interpreted cautiously. The categories "Others" in the "City of residence" variable and "Other" in the "Employment status" variable encompass various less frequent subcategories.

Variable	Category	Count	Percentage
Age (years)	18-24	581	33.0%
	25-34	341	19.3%
	35-44	246	14.0%
	45-54	311	17.6%
	55-64	215	12.2%
	Above 65	21	1.2%
	Under 18	48	2.7%
Gender	Female	1052	59.7%
	Male	711	40.3%
Education level	Bachelor’s degree	1216	69.0%
	Diploma	92	5.2%
	Doctorate or higher	26	1.5%
	High school or less	317	18.0%
	Master’s degree	112	6.4%
Employment status	Employed full-time	655	37.2%
	Employed part-time	50	2.8%
	Other	86	4.9%
	Retired	205	11.6%
	Student	545	30.9%
	Unemployed	222	12.6%
City of residence	Eastern province	578	32.8%
	Middle province	730	41.4%
	Northern province	53	3.0%
	Others	17	1.0%
Geographic location	South province	21	1.2%
	Western province	364	20.6%
	Rural	46	2.6%
	Suburban	104	5.9%
	Urban	1613	91.5%

General information associated with the general health of the participants

Our survey investigated various factors related to physical exercise, smoking, alcohol consumption, mobility and balance difficulties, overall health status, fall occurrence, and injuries among older adults (Table 2). Most respondents reported engaging in physical exercise either rarely or never (42.1%) or several times a week (29.7%). Smoking prevalence was relatively low, with 18.9% of participants reporting current smoking. Alcohol consumption was rare, with 96.5% indicating rarely or never consuming alcohol. A significant portion of respondents (19.6%) reported experiencing difficulties with mobility or balance. Most respondents rated their overall health status as good (42.2%), followed by excellent (40.0%). In the past year, 31.7% of older adults reported experiencing falls occasionally, while 58.3% reported rare occurrences. Among these incidents, fractures were identified as the most prevalent injury, accounting for 44.5% of fall-related injuries. It is significant to note that 91.3% of participants acknowledged that falls often led to injuries among the elderly. Of those surveyed, 31.7% received fall prevention interventions or support, which included measures such as balance training, home modifications, and the use of assistive devices.

**Table 2:** General information of the study participants The data presented in the table is based on responses from a sample of older adults and may not represent the entire population. Percentages may not add up to exactly 100% due to rounding. The categories "Several times a week" and "Occasionally" in the "How often do you engage in regular physical exercise?" variable had relatively higher counts and should be interpreted with greater significance. The categories "Rarely or never" in the "How often do you consume alcohol?" variable and "None" in the "How frequently have falls occurred in the past year?" variable had notably higher counts, indicating low alcohol consumption and falls occurrences, respectively.

Variable	Category	Count	Percentage
How often do you engage in regular physical exercise?	Daily	134	7.6%
	Once a week	364	20.6%
	Rarely or never	742	42.1%
	Several times a week	523	29.7%
Do you currently smoke?	No	1430	81.1%
	Yes	333	18.9%
How often do you consume alcohol?	Daily	22	1.2%
	Occasionally	40	2.3%
	Rarely or never	1701	96.5%
Do you experience any difficulties with mobility or balance?	No	1418	80.4%
	Yes	345	19.6%
How would you rate your overall health status?	Excellent	706	40.0%
	Fair	296	16.8%
	Good	744	42.2%
	Poor	17	1.0%
How frequently have falls occurred in the past year?	Frequently (5 or more)	17	1.0%
	None	1027	58.3%
	Occasionally (3-4 falls)	160	9.1%
	Rarely (1-2 falls)	559	31.7%
Have you had any fractures or injuries as a result of a fall?	No	1476	83.7%
	Yes	287	16.3%
Have falls resulted in any injuries among older adults?	No	154	8.7%
	Yes	1609	91.3%

Chronic diseases and medications

We surveyed various health-related variables among the individuals. Around 74% reported having no chronic health conditions, while 5.3% had one, and 3.6% had two chronic health conditions (Table 3). A smaller proportion, 4.2%, indicated having three or more chronic health conditions. Hypertension was diagnosed in 17.5% of participants, while 13.6% had been diagnosed with diabetes. Arthritis affected 13.7% of the respondents, and 6.1% had a diagnosis of heart disease. Almost a third of the participants (29.9%) were taking medications for chronic health conditions, with 29.8% on one to three medications, and only 1.4% on seven or more. Side effects from medications were experienced by 19.7% respondents, and 9.5% reported falling while taking medications. These findings shed light on the prevalence of chronic conditions and medication-related issues within the surveyed population.

**Table 3:** Descriptives of chronic diseases and medications The table presents health-related findings among the surveyed individuals, including chronic conditions, medication usage, and fall occurrences. Caution is advised for unusual categories, and the data reflects a sample of respondents.

Variable	Category	Count	Percentage
How many chronic health conditions do you currently have?	1	93	5.3%
	2	63	3.6%
	3	74	4.2%
	4	239	13.6%
	5	165	9.4%
	None	1533	74.0%
Are you currently diagnosed with hypertension?	No	1455	82.5%
	Yes	308	17.5%
Have you been diagnosed with diabetes?	No	1523	86.4%
	Yes	240	13.6%
Do you have arthritis?	No	1521	86.3%
	Yes	242	13.7%
Have you been diagnosed with heart disease?	No	1655	93.9%
	Yes	108	6.1%
Are you taking medications for any chronic health conditions?	No	1235	70.1%
	Yes	528	29.9%
How many medications are you currently taking?	1-3	526	29.8%
	4-6	128	7.3%
	7 or more	25	1.4%
	None	1084	61.5%
Do you experience any side effects from your medications?	No	1415	80.3%
	Yes	348	19.7%
Have you ever fallen while taking medications?	No	1596	90.5%
	Yes	167	9.5%

Falls in adults

The majority reported no frequent falls (1,027, 58.3%) or fractures resulting from falls (1,476, 83.7%). However, falls caused injuries among a significant proportion of older adults (1,609, 91.3%); see Table 4. Interestingly, only 499 (31.7%) participants received fall prevention interventions or support. The data also highlights aspects such as lighting arrangements, presence of non-slip surfaces, tripping hazards, and sufficient space for mobility aids in the home. These findings underscore the importance of fall prevention strategies and potential areas for improvement in the living environment.

**Table 4:** Falls in adults Data is presented as numbers and percentages. Percentages may not add up to 100% due to rounding.

Variable	Category	Count
Frequency of falls in the past year	Frequently (5 or more)	17
	None	1027
	Occasionally (3-4 falls)	160
	Rarely (1-2 falls)	559
Fractures or injuries from falls	No	1476
	Yes	287
Injuries resulting from falls among older adults	No	154
	Yes	1609
Current receipt of fall prevention interventions or support	No	1204
	Yes	559
Falls while taking medications	No	1596
	Yes	167
Environmental hazards contributing to falls	No	1270
	Yes	493
Adequate lighting in living space	No	239
	Not sure	170
	Yes	1354
Non-slip surfaces or mats in moisture-prone areas	No	770
	Not applicable	137
	Yes	856
Tripping hazards in living space	No	1127
	Yes	636
Sufficient space for mobility aids in home	No	617
	Not applicable	172
	Yes	974
Received education on fall prevention strategies	No	1373
	Yes	390

Our analysis of falls in adults revealed interesting associations with various demographic factors (Table 5). Age showed no significant difference in fall occurrences (p = 0.252), though individuals under 18 experienced higher fall rates (38, 79.2%). Gender displayed a significant association, with females experiencing more falls (501, 47.6%) compared to males (235, 33.1%) (p < 0.001).

**Table 5:** Association between adult falls and sociodemographic factors p-values were calculated using appropriate statistical tests to determine the significance of associations between demographic factors and falls in adults. Significant associations are indicated by p < 0.05, highlighting the relevance of gender, education level, employment status, geographic location, and alcohol consumption in predicting fall occurrences among adults.

		Falls in adults				
		No		Yes		p-value
		Count	Percentage	Count	Percentage	
Age (years)	18-24	357	61.4%	224	38.6%	0.252
	25-34	203	59.5%	138	40.5%	
	35-44	160	65.0%	86	35.0%	
	45-54	163	52.4%	148	47.6%	
	55-64	120	55.8%	95	44.2%	
	Above 65	14	66.7%	7	33.3%	
	Under 18	10	20.8%	38	79.2%	
Gender	Female	551	52.4%	501	47.6%	<0.001
	Male	476	66.9%	235	33.1%	
Education level	Bachelor’s degree	766	63.0%	450	37.0%	<0.001
	Diploma	47	51.1%	45	48.9%	
	Doctorate or higher	10	38.5%	16	61.5%	
	High school or less	129	40.7%	188	59.3%	
	Master’s degree	75	67.0%	37	33.0%	
Employment status	Employed full-time	430	65.6%	225	34.4%	<0.001
	Employed part-time	28	56.0%	22	44.0%	
	Other	31	36.0%	55	64.0%	
	Retired	117	57.1%	88	42.9%	
	Student	310	56.9%	235	43.1%	
	Unemployed	111	50.0%	111	50.0%	
Geographic location	Rural	32	69.6%	14	30.4%	0.326
	Suburban	65	62.5%	39	37.5%	
	Urban	930	57.7%	683	42.3%	
How often do you engage in regular physical exercise?	Daily	84	62.7%	50	37.3%	0.252
	Once a week	177	48.6%	187	51.4%	
	Rarely or never	459	61.9%	283	38.1%	
	Several times a week	307	58.7%	216	41.3%	
Do you currently smoke?	No	808	56.5%	622	43.5%	0.07
	Yes	219	65.8%	114	34.2%	
How often do you consume alcohol?	Daily	12	54.5%	10	45.5%	0.04
	Occasionally	15	37.5%	25	62.5%	
	Rarely or never	1000	58.8%	701	41.2%	

Association between falls, chronic diseases and medications

Our analysis revealed significant associations between various health conditions and medication usage and the occurrence of falls. Participants diagnosed with hypertension showed a higher percentage of falls (165, 53.6%) compared to those without hypertension (571, 39.2%) (p < 0.001). Similarly, individuals with diabetes had a significantly higher percentage of falls (120, 50.0%) compared to those without diabetes (614, 40.4%) (p < 0.001). Table 6 demonstrates the factors associated with increased falls in adults.

**Table 6:** Association between falls, chronic diseases and medications p-values were calculated using appropriate statistical tests to determine the significance of associations between variables and falls in older adults. Significant associations are indicated by p < 0.001, highlighting the relevance of various health conditions and medication usage in predicting the occurrence of falls in the emergency room setting in Saudi Arabia.

		Falls in adults				p-value
		No		Yes		
		Count	Percentage	Count	Percentage	
Are you currently diagnosed with hypertension?	No	884	60.8%	571	39.2%	<0.001
	Yes	143	46.4%	165	53.6%	
Have you been diagnosed with diabetes?	No	907	59.6%	616	40.4%	<0.001
	Yes	120	50.0%	120	50.0%	
Do you have arthritis?	No	907	59.6%	614	40.4%	<0.001
	Yes	120	49.6%	122	50.4%	
Have you been diagnosed with heart disease?	No	966	58.4%	689	41.6%	0.125
	Yes	61	56.5%	47	43.5%	
Are you taking medications for any chronic health conditions?	No	777	62.9%	458	37.1%	<0.001
	Yes	250	47.3%	278	52.7%	
How many medications are you currently taking؟	1-3	273	51.9%	253	48.1%	<0.001
	4-6	54	42.2%	74	57.8%	
	7 or more	11	44.0%	14	56.0%	
	None	689	63.6%	395	36.4%	
Do you experience any side effects from your medications?	No	887	62.7%	528	37.3%	<0.001
	Yes	140	40.2%	208	59.8%	

Multivariate analysis of factors that predict falls in adults

Significant associations were found between falls and several factors, including gender (female), education level (master's degree), employment status (other, retired, student), and alcohol consumption (occasionally) (Table 7). However, no significant associations were found with age and geographic location. These findings provide valuable insights into the risk factors for falls in older adults, highlighting the importance of targeted preventive measures and interventions to reduce fall-related incidents in this vulnerable population.

**Table 7:** Multivariate analysis of factors that predict falls in adults The logistic regression analysis revealed significant associations between falls in adults and gender (female), education level (master's degree), employment status (other, retired, student), and alcohol consumption (occasionally). Other factors, such as age and geographic location, were not statistically significant predictors of falls in this analysis. The odds ratios and 95% confidence intervals provide insights into the strength and direction of the association.

Variable	Odds ratio	95% confidence interval	p-value
Gender (reference: female)	1.54	1.20-1.98	<0.001
Education level			<0.001
High school or less	1 (reference)		
Diploma	0.92	0.68-1.25	
Bachelor’s degree	0.78	0.61-1.01	
Master’s degree	0.60	0.42-0.85	
Employment status			<0.001
Employed full-time	1 (reference)		
Employed part-time	1.17	0.74-1.85	
Other	2.21	1.44-3.40	
Retired	1.65	1.26-2.17	
Student	1.32	1.06-1.64	
Unemployed	1.14	0.88-1.47	
Geographic location			0.326
Urban	1 (reference)		
Rural	1.32	0.85-2.04	
Suburban	0.98	0.74-1.30	
Alcohol consumption			0.04
Rarely or never	1 (reference)		
Occasionally	1.68	1.10-2.57	
Daily	1.12	0.61-2.05	

Admission to the ER after falls and satisfaction with the ER

The respondents rated various aspects of the ER staff's services, including responsiveness, clarity and effectiveness of communication, involvement in decision-making, coordination of care, and likelihood of recommending the ER to others. Overall, the majority of respondents expressed positive views, with a significant percentage rating the ER staff as "Excellent" or "Above average" (Table 8). These findings highlight the generally satisfactory quality of care provided by the ER staff. However, some areas, such as communication and decision-making involvement, received relatively lower ratings, indicating areas that may require improvement to enhance patient experiences.

**Table 8:** Admission to the ER after falls and satisfaction with the ER ER, emergency room

		Count	%
How would you rate the responsiveness of the ER staff in attending to your needs promptly?	Above average	367	20.8%
	Average	533	30.2%
	Below average	114	6.5%
	Excellent	696	39.5%
	Poor	53	3.0%
How satisfied are you with the clarity and effectiveness of communication from the ER staff regarding your condition and treatment option?	Above average	381	21.6%
	Average	550	31.2%
	Below average	92	5.2%
	Excellent	696	39.5%
	Poor	44	2.5%
To what extent did the ER staff involve you in decision-making regarding your treatment plan?	Above average	382	21.7%
	Average	564	32.0%
	Below average	122	6.9%
	Excellent	638	36.2%
	Poor	57	3.2%
How satisfied are you with the overall coordination of care provided by the ER staff, including referrals to specialists or follow-up care?	Above average	399	22.6%
	Average	533	30.2%
	Below average	89	5.0%
	Excellent	687	39.0%
	Poor	55	3.1%
How likely are you to recommend the ER to family or friends based on your experience?	likely	468	26.5%
	Neutral	415	23.5%
	Not likely at all	93	5.3%
	Slightly likely	85	4.8%
	Very	702	39.8%

## Discussion

Analysis and implications

Falls in older adults can be attributed to various factors. Among them, physiological changes associated with aging can impact balance and gait [6]. Medical conditions such as Parkinson's disease and stroke also play a significant role [7]. Moreover, the use of certain medications, especially antihypertensives, has been identified as a potential contributor [8]. It is worth noting that while the United Nations provides a clear definition of older adults [9], this study specifically categorized individual ages (specific age or range), which may vary from other definitions. Given the prevalent incidence of hypertension among older populations, it is essential to critically evaluate the correlation between these factors [10-12]. We aim to provide a holistic understanding of the various determinants contributing to falls in the elderly. Consequences of falls are multifaceted, from physical injuries to psychological impacts like depression and social withdrawal [13]. With the growing global aging population, the urgency for effective prevention strategies, including physical activity promotion and medication monitoring, is evident [14].

Within healthcare settings, falls compromise patient safety. Major risk factors for falls in elderly patients include age, especially beyond 65 years, and gender, with females being more susceptible [15-17]. The implications of falls are wide-ranging, from fractures and head injuries to prolonged hospitalizations [18]. In the Middle East, falls are a significant cause of morbidity, placing financial burdens on healthcare [19]. Hospital data shows that children and women are at an elevated risk [2,16-20]. Saudi Arabia, specifically, has seen nearly half of its elderly population experiencing at least one fall in the past year, emphasizing the interplay between age, health disorders, and fall risks [21].

In our study, descriptive statistics revealed demographic patterns, and the chi-square test assessed associations between falls and varied factors. Logistic regression helped identify independent fall risk determinants, and a perception-driven survey evaluated ER patient satisfaction.

Insights from results

The study showcased a pronounced representation within the age groups of 18-24 and 25-34, underlining the need to address both younger and older populations. The diverse gender and educational backgrounds of the participants highlight the importance of a holistic intervention approach. Notably, limited physical activity and infrequent alcohol consumption were dominant, suggesting areas for health promotion. Moreover, the prevalence of mobility challenges stresses the urgency for therapeutic interventions. These insights can be pivotal for crafting preventive strategies catered to specific risk determinants.

Future research avenues

A promising direction is to delve deeper into the interplay of cultural, regional, and socioeconomic factors with fall incidents. By understanding how these aspects uniquely impact different demographic segments, we can tailor interventions accordingly. Also, broadening our study population is essential. By encompassing diverse backgrounds and regions, our findings will achieve greater applicability, offering insights that can be personalized for various elderly communities.

While the study is valuable, it is crucial to acknowledge its inherent limitations, such as reliance on self-reported data and the potential for recall bias due to its cross-sectional design. Also, the potential lack of comprehensive representation from varied socioeconomic and cultural backgrounds could impact the generalizability of our findings to broader populations. Addressing this in subsequent research would further validate our conclusions and recommendations.

By focusing on these areas for future research, we can better comprehend the intricacies of fall prevention and emergency care for the elderly. Recognizing the significance of cultural, regional, and socioeconomic nuances will enable the development of more targeted, effective interventions tailored to the elderly population's diverse needs.

## Conclusions

Falls, especially among the elderly, are a significant global health concern, with the elderly, and females in particular, being the most vulnerable. In the Middle East, including Saudi Arabia, the severe consequences of falls, from fractures to lacerations, emphasize the need for targeted preventive measures. Our study within Saudi emergency departments offers insights into fall predictors, underscoring age, gender disparities, and specific health factors as crucial determinants. Encouragingly, feedback on emergency care quality remains positive, although improvements in communication and patient involvement in decision-making are needed.

Based on our findings, we advocate for proactive strategies in Saudi Arabia, focusing on identifying high-risk individuals, enhancing healthcare support, and improving patient-caregiver interactions. Implementing interventions at homes, emphasizing safety, monitoring medication side effects, and assessing fracture risks can significantly mitigate fall occurrences. These measures are vital for the well-being and quality of life of Saudi Arabia's citizens, especially the elderly, paving the way for a safer, healthier future.

## Appendices

Questionnaire used in the research

**Table 9:** Questionnaire for the comprehensive health and environmental survey on falls among older adults

Questionnaire	
Section 1: Demographics	
Question	Options
Age	A. Under 18
	B. 18-24
	C. 25-34
	D. 35-44
	E. 45-54
	F. 55-64
	G. 65 or above
Gender	A. Male
	B. Female
Education level	A. High school or less
	B. Diploma
	C. Bachelor's degree
	D. Master's degree
	E. Doctorate or higher
Employment status	A. Employed full-time
	B. Employed part-time
	C. Unemployed
	D. Student
	E. Retired
	F. Other
City of residence	A. Middle Province
	B. Eastern Province
	C. Northern Province
	D. South Province
	E. Western Province
	F. Others
Geographic location	A. Urban
	B. Suburban
	C. Rural
Section 2: General questions	
Question	Options
How often do you engage in regular physical exercise?	A. Daily
	B. Several times a week
	C. Once a week
	D. Rarely or never
Do you currently smoke?	A. Yes
	B. No
How often do you consume alcohol?	A. Daily
	B. Occasionally
	C. Rarely or never
Are you currently taking any medications?	A. Yes
	B. No
Do you experience any difficulties with mobility or balance?	A. Yes
	B. No
How would you rate your overall health status?	A. Excellent
	B. Good
	C. Fair
	D. Poor
How frequently have falls occurred in the past year?	a) None
	b) Rarely (1-2 falls)
	c) Occasionally (3-4 falls)
	d) Frequent (5 or more falls)
Have you had any fractures or injuries as a result of a fall?	A. Yes
	B. No
Which of the following factors do you believe contribute to falls among older adults? (Select all that apply)	a) Poor balance and coordination
	b) Medications affecting balance or causing dizziness
	c) Vision problems or difficulty seeing clearly
	d) Muscle weakness or decreased strength
	e) History of previous falls
	f) Dizziness or lightheadedness
	g) Environmental hazards (e.g., uneven surfaces, poor lighting)
	h) Lack of regular exercise or physical activity
Have falls resulted in any injuries among older adults?	a) Yes
	b) No
If yes, please select the most common type of injury sustained. (Select one)	a) Fractures
	b) Sprains or strains
	c) Head injury or concussion
	d) Bruises or contusions
	e) Other
Are you or the person you know currently receiving any fall prevention interventions or support?	a) Yes
	b) No
If yes, please specify the type of interventions or support received. (Select all that apply)	a) Physical therapy or exercise programs
	b) Home modifications for safety (grab bars, handrails, etc.)
	c) Medication review or adjustment
	d) Vision assessment or correction
	e) Use of mobility aids (cane, walker, etc.)
	f) Other
Section 3: Correlation between chronic health conditions, medication use, and falls	
Question	Options
How many chronic health conditions do you currently have?	A. None
	B. 1
	C. 2
	D. 3 or more
Are you currently diagnosed with hypertension?	A. Yes
	B. No
Have you been diagnosed with diabetes?	A. Yes
	B. No
Do you have arthritis?	A. Yes
	B. No
Have you been diagnosed with heart disease?	A. Yes
	B. No
Are you taking medications for any chronic health conditions?	A. Yes
	B. No
How many medications are you currently taking?	A. None
	B. 1-3
	C. 4-6
	D. 7 or more
Do you experience any side effects from your medications?	A. Yes
	B. No
Have you ever fallen while taking medications?	A. Yes
	B. No
Section 4: Environmental factors and fall outcomes in older adults presenting to the emergency room	
Question	Options
Are there any environmental hazards in your home that may contribute to falls?	A. Yes
	B. No
Are there adequate lighting arrangements in your living space?	A. Yes
	B. No
	C. Not sure
Is your home equipped with non-slip surfaces or mats in areas prone to moisture (e.g., bathroom, kitchen)?	A. Yes
	B. No
	C. Not applicable
Are there any tripping hazards (e.g., loose rugs, cluttered pathways) present in your living space?	A. Yes
	B. No
Does your home have sufficient space for maneuvering mobility aids (e.g., walkers, wheelchairs)?	A. Yes
	B. No
	C. Not applicable
Have you received any education or guidance on fall prevention strategies?	A. Yes
	B. No
Section 5: Quality of ER intervention	
Question	Options
How would you rate the responsiveness of the ER staff in attending to your needs promptly?	(1) Poor, (2) Below average, (3) Average, (4) Above average, (5) Excellent
How satisfied are you with the clarity and effectiveness of communication from the ER staff regarding your condition and treatment options?	(1) Poor, (2) Below average, (3) Average, (4) Above average, (5) Excellent
To what extent did the ER staff involve you in decision-making regarding your treatment plan?	(1) Poor, (2) Below average, (3) Average, (4) Above average, (5) Excellent
How satisfied are you with the overall coordination of care provided by the ER staff, including referrals to specialists or follow-up care?	(1) Poor, (2) Below average, (3) Average, (4) Above average, (5) Excellent
How likely are you to recommend the ER to family or friends based on your experience?	(1) Not likely at all, (2) Slightly likely, (3) Neutral, (4) Likely, (5) Very likely
